# Ultrasound-guided injection of ankle contouring with botulinum neurotoxin

**DOI:** 10.1016/j.jpra.2025.11.034

**Published:** 2025-12-12

**Authors:** Kyu-Ho Yi, Jin-Hyun Kim, Jong-Keun Song, Jeremy B. Green, Thomas Rappl, Michael H. Gold, Jeongho Sohn, Benjamin Ascher, Roya Zarmehr Zamin, Rosa Sigrist, Ximena Wortsman

**Affiliations:** a You and I Clinic, Seoul, Republic of Korea; b Division in Anatomy and Developmental Biology, Department of Oral Biology, Human Identification Research Institute, BK21 FOUR Project, Yonsei University College of Dentistry, Seoul, Republic of Korea; c Pixelab Plastic Surgery Clinic, Seoul, Republic of Korea; d Skin Associates of South Florida/Skin Research Institute Coral Gables, FL, USA; e Medical Aesthetic Research Academy, Graz, Austria; f The Tennessee Clinical Research Center, Nashville, TN, USA; g College of Medicine, Korea University, MSc Program in Clinical Biomedical Engineering, Seoul, Republic of Korea; h SiBUS Inc., Paris, France; i Dentofaces Clinic, Dubai, UAE; j DRZMEDAESTHETICS Academy (Medical Aesthetics Training Center), Dubai, UAE; k Department of Radiology, Faculty of Medicine, University of São Paulo, São Paulo, Brazil; l Department of Dermatology, Faculty of Medicine, Universidad de Chile, Santiago, Chile; m Department of Dermatology, School of Medicine, Pontificia Universidad Catolica de Chile, Santiago, Chile

**Keywords:** Ankle contouring, Botulinum toxin injection, Ultrasound guidance, Soleus muscle atrophy, Minimally invasive procedure

## Abstract

**Background:**

A slim ankle is considered a hallmark of beauty in modern aesthetics. Excessive muscular hypertrophy, particularly of the deep calf muscle (soleus), can lead to a thick ankle appearance that detracts from an overall refined leg contour. Although surgical approaches have been used historically, they carry risks such as scarring, contracture, and unpredictable outcomes.

**Objective:**

This study aimed to evaluate the efficacy and safety of ultrasound-guided botulinum toxin type A (JETEMA THE TOXIN, JETEMA Co., Ltd. Korea) injections for improving ankle contour in patients with thick ankles.

**Methods:**

Three adult female patients with noticeably hypertrophic calf muscles underwent ultrasound-guided injection of 20 units of botulinum toxin into the soleus muscles. Needles were inserted with ultrasound guidance to ensure precise real-time visualization and accurate injection into the soleus muscles. Ankle circumference and soleus muscle thickness were measured pre-treatment and at 8 weeks post-treatment using standardized methods. Wilcoxon signed-rank test was applied to evaluate statistical significance.

The manuscript was checked against the Strengthening the Reporting of Observational Studies in Epidemiology (STROBE) checklist (Supplemental Appendix).

**Results:**

All patients exhibited a reduction in ankle circumference (approximately 6.7–7.1 %) and soleus muscle thickness (around 14–15 %) at the 8-week follow-up. The Wilcoxon test revealed statistically significant differences (*p* = 0.001) between pre- and post-treatment values. Visual assessment of photographic records further confirmed a noticeably slimmer and more refined ankle contour.

**Conclusion:**

Ultrasound-guided botulinum toxin injections appear to be a safe and effective minimally invasive approach for ankle contouring, achieving clinically meaningful reductions in muscle bulk with high patient satisfaction.

## Introduction

A slim ankle is widely regarded as a standard of beauty in modern aesthetics. Since the ankle region has a very thin layer of subcutaneous fat, muscular development directly influences ankle circumferenc.^1^ In particular, hypertrophy of the soleus, a deep calf muscle, is a principal cause of thick ankles, making reduction in the soleus muscle volume critical for improving ankle contours.^2^ Although procedures such as selective neurectomy and radiofrequency muscle reduction have traditionally been used to correct thick ankles, these surgical approaches carry risks of ankle joint contracture, painful scarring, and uncertainty regarding their outcomes.^3^, ^4^, ^5^

In contrast, botulinum toxin injections into muscle can selectively induce paralysis, reducing muscular activity and gradually causing muscle atrophy. Compared with surgery, this approach is considered relatively safe and minimally invasive, imposing less disruption on daily life.^1^^,^^6^ Botulinum toxin type A, in particular, has been extensively utilized for facial wrinkle treatments, and more recently, its indications have expanded to diverse cosmetic applications, including the correction of neck and shoulder lines, yet no research has been conducted specifically on improving ankle contours.^7^, ^8^, ^9^

Ultrasound-guided injection offers the advantage of real-time visualization and differentiation between gastrocnemius and soleus muscles with precise needle placement in the soleus muscle.^10^^,^^11^ The technique can minimize damage to surrounding nerves and vessels but achieve sufficient outcomes with lower toxin doses.^12^

While volume reduction of soleus muscle is well studied from previous research, there has been limited research on botulinum toxin injections for ankle contouring, especially those employing targeted methods with ultrasound guidance.^13^ Therefore, this study aims to examine the efficacy of an ultrasound-guided botulinum toxin injection protocol for patients having thick ankles, specifically by measuring reductions in ankle circumference and improvements in overall contour. We hope to broaden the utilization of minimally invasive procedures in ankle contour correction and contribute to the development of systematic ultrasound-guided botulinum toxin injection guidelines.

## Materials and methods

### Study participants

Our study included three adult female patients who wished to enhance the cosmetic appearance of their ankles. All of them presented with noticeably hypertrophic calf muscles around the ankle and complained about their calves. None had any underlying neurological conditions or symptoms, lower-limb edema, or other relevant medical issues. Before proceeding with treatment each patient was thoroughly briefed on the procedure, expected outcomes, potential risks, and provided informed consent.^14^

### Procedure

The procedure was performed with the patient seated and a topical anesthetic cream applied, ensuring that the patient’s ankle remained in as much extension as possible. If necessary, a medical staff member supported the patient’s foot gently to allow stable exposure of the injection site. Botulinum toxin type A (JETEMA THE TOXIN, JETEMA Co., Ltd. Korea) was used, prepared by diluting one vial (100 units) in 3 mL of normal saline.^15^

**Figure 1:**
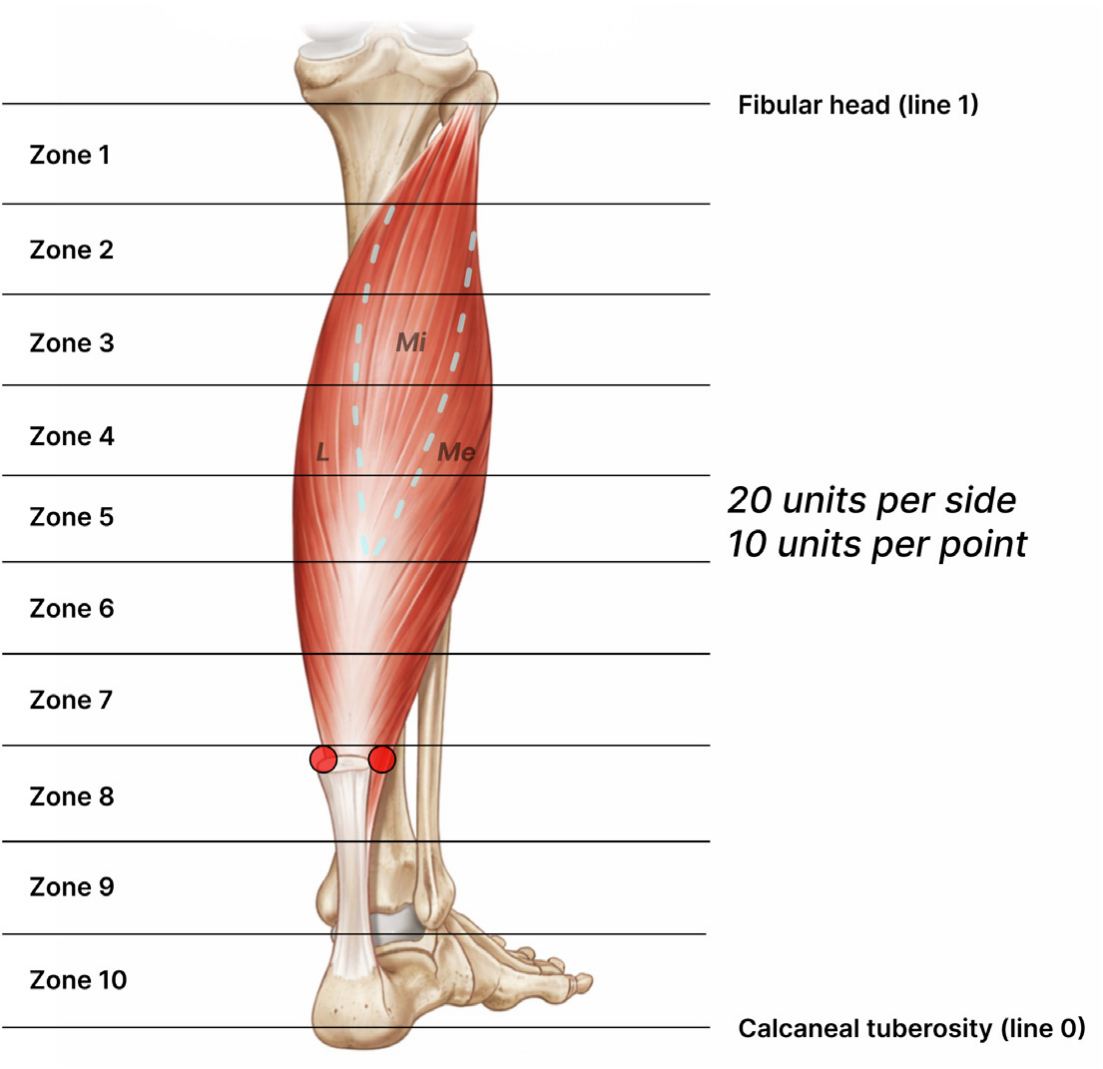
Anatomical compartment (zone) classification of the calf muscles. The segment from right below the knee to the distal calf was divided into ten equal sections, with the fibular head (line 1) designated as the superior landmark and the calcaneal tuberosity (line 0) as the inferior landmark. This diagram illustrates the anatomical relationship between the soleus muscle and the Achilles tendon, highlighting the zones corresponding to the mid-regions of the muscle.

**Figure 2:**
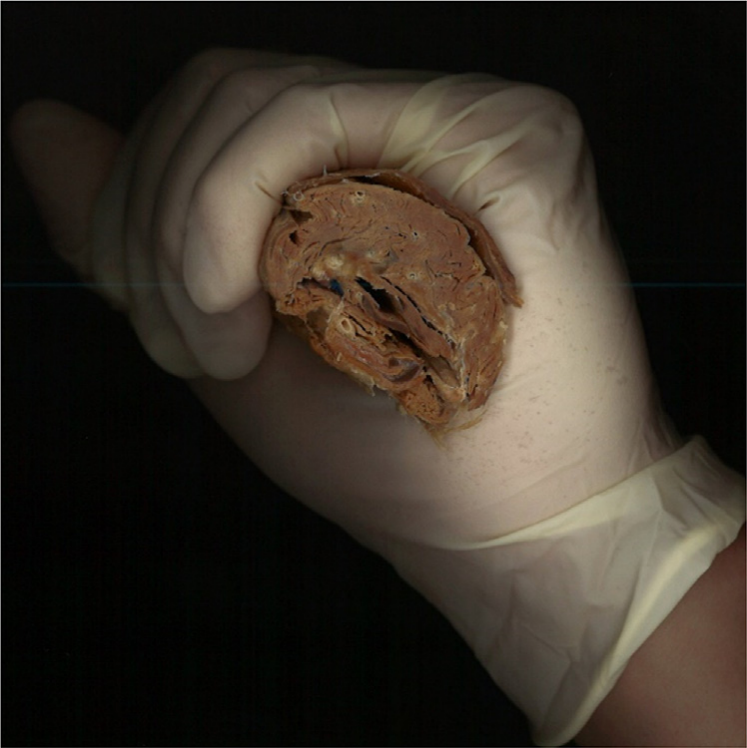
A cross-sectional specimen taken 10 cm above the medial malleolus, showing the thickness of the soleus muscle. This is the area targeted for botulinum toxin injection.

**Figure 3:**
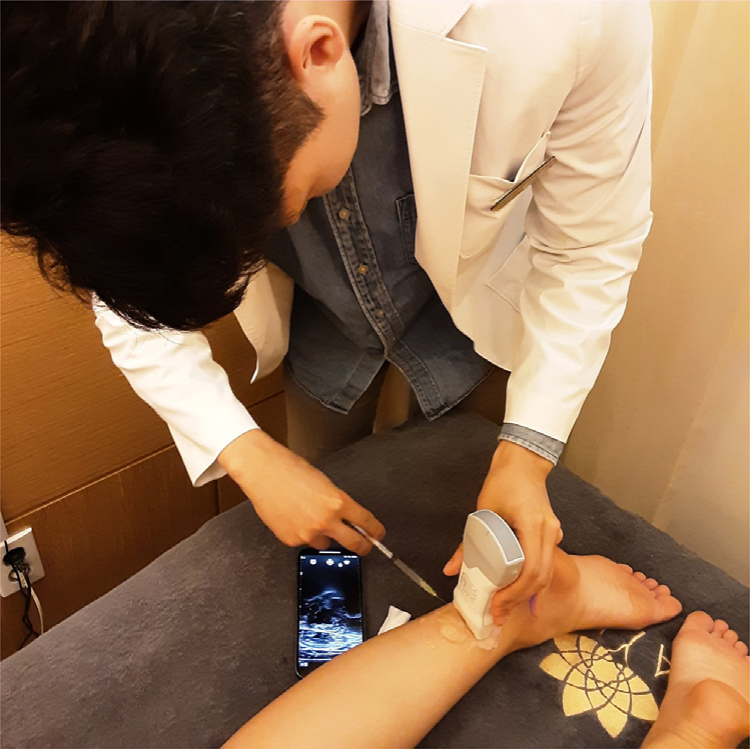
The Vscan wireless handheld ultrasound device, along with a 1 mL syringe and a 27-gauge needle.

**Figure 4:**
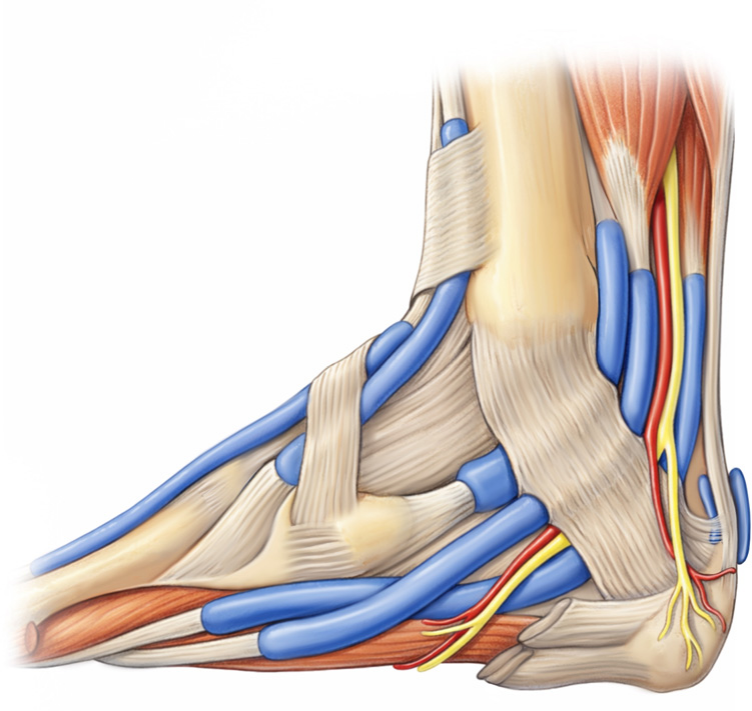
An illustration of the musculoskeletal structure in the ankle region.

**Figure 5:**
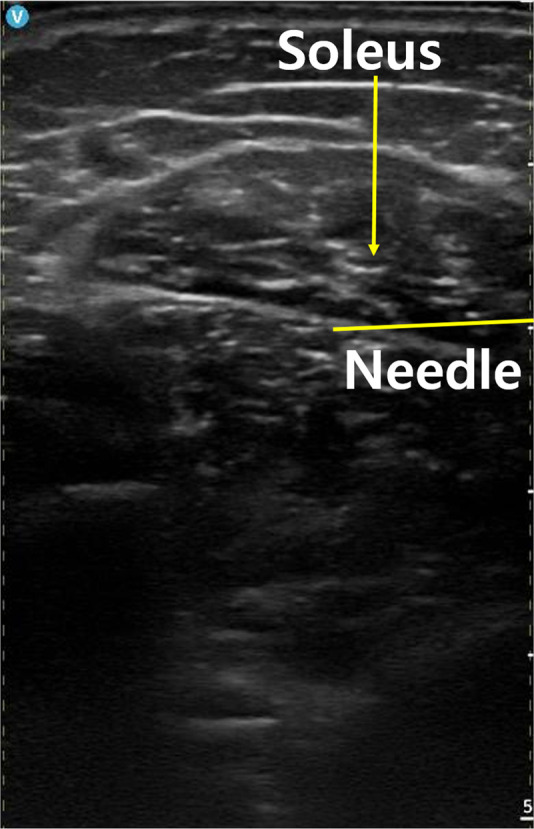
An ultrasound image depicting the cross-sectional view of the injection site.

A 27 G needle for intramuscular injection was inserted almost parallel to the ultrasound probe, allowing the tip to be continuously visualized in real-time monitoring display. About 3–12 MHz high-frequency linear probe and wireless handheld ultrasonography system (Vscan, GE, USA) were used, with medical gel for ultrasound applied to both the probe and the patient’s skin for optimal imaging quality.^16^ All procedures were performed by two trained physicians using the same proportional landmark method (30 % distance from the medial malleolus to the fibular head). Repeated measurements were compared to minimize inter-observer variability in landmark identification. 20 units of botulinum toxin, 10 units for medial and 10 units for lateral sides, were injected into the zone 8, the area about 10 cm above the medial malleolus (Figures 1, 2). During the procedure, we used ultrasound monitoring to ensure that the needle tip to avoid the Achilles tendon, blood vessels, and nerves, thus accurately delivering the toxin into the soleus muscle (Figure 3, Figure 4, Figure 5).

## Results

Using the method described above, three patients underwent ultrasound-guided botulinum toxin injections. All three demonstrated reductions in ankle circumference and improvements in contour following the procedure. At the 8-week follow-up:

In Patient 1 (32-year-old female), the right ankle circumference decreased from 21.8 cm before treatment to 20.3 cm, a 1.5 cm (approximately 6.9 %) reduction. Ultrasound measurement of the soleus muscle showed a decrease from 13.8 mm to 11.7 mm (about 15.2 %). On the left side, the circumference declined from 22.2 cm to 20.7 cm (1.5 cm, 6.8 %), with soleus muscle thickness reducing from 13.6 mm to 11.5 mm (approximately 15.4 %).

In Patient 2 (39-year-old female), the right ankle circumference dropped from 22.7 cm to 21.1 cm (1.6 cm, about 7.0 %), and muscle thickness decreased from 14.5 mm to 12.3 mm (roughly 15.2 %). On the left, the circumference reduced from 22.4 cm to 20.8 cm (1.6 cm, around 7.1 %), and muscle thickness declined from 14.3 mm to 12.2 mm (about 14.7 %).

In Patient 3 (46-year-old female), the right ankle circumference went from 23.0 cm to 21.4 cm (1.6 cm, 7.0 %), and the soleus thickness reduced from 14.8 mm to 12.6 mm (about 14.9 %). The left ankle showed a reduction in circumference from 22.5 cm to 21.0 cm (1.5 cm, 6.7 %), with muscle thickness declining from 14.6 mm to 12.5 mm (approximately 14.4 %).

All three patients demonstrated consistent reductions in ankle circumference (approximately 6.7–7.1 %) and soleus muscle thickness (around 14–15 %). While the small sample size limits generalizability, each patient experienced a clinically meaningful improvement. A Wilcoxon signed-rank test, suitable for small sample sizes, revealed a statistically significant result (*p* = 0.001). The mean reduction in ankle circumference was 7.0 % (95 % confidence interval [CI], 5.8–8.2 %), and the reduction in soleus muscle thickness was 14.8 % (95 % CI, 13.4–16.1 %). In addition to the Wilcoxon signed-rank test, paired *t*-tests were performed, yielding consistent significance levels (*p* < 0.001). Despite being preliminary, these findings suggest that ultrasound-guided botulinum toxin injections can effectively reduce ankle girth and muscle volume in patients with bulky ankles. Visual comparison of pre- and post-treatment photographs revealed notably slimmer and more refined contours (Table 1).

**Table 1:** Changes in ankle circumference and muscle thickness in three patients.

Patient (age)	Side	Ankle circumference (cm)			Soleus thickness (mm)		
		Pre	Post	Change (%)	Pre	Post	Change (%)
Patient 1 (32)	Right	21.8	20.3	1.5 (6.9 %)	13.8	11.7	2.1 (15.2 %)
	Left	22.2	20.7	1.5 (6.8 %)	13.6	11.5	2.1 (15.4 %)
Patient 2 (39)	Right	22.7	21.1	1.6 (7.0 %)	14.5	12.3	2.2 (15.2 %)
	Left	22.4	20.8	1.6 (7.1 %)	14.3	12.2	2.1 (14.7 %)
Patient 3 (46)	Right	23.0	21.4	1.6 (7.0 %)	14.8	12.6	2.2 (14.9 %)
	Left	22.5	21.0	1.5 (6.7 %)	14.6	12.5	2.1 (14.4 %)

### Mid- and long-term follow-up on adverse events

Mild pain at the injection site was reported the day after the procedure, but it resolved spontaneously without analgesics. In cases where minor bruising occurred, it disappeared within 2 weeks, and no infections, inflammatory reactions, or other serious complications were observed. At the 8-week follow-up, patients did not complain of weakness, gait disturbances, paresthesia, or any impairment in their ankle range of motion. Additional follow-up conducted up to 12 weeks post-treatment revealed no further complications or excessive muscle atrophy. All subjects expressed satisfaction with the ankle contouring results. Although long-term maintenance of the outcomes may depend on additional procedures or further monitoring, these findings indicate a clear, short-term improvement in ankle contours and confirm that ultrasound-guided botulinum toxin injections are both safe and effective, with no significant adverse events or functional impairments observed through mid- and long-term follow-up. A time-course analysis of ankle circumference and soleus muscle thickness from baseline to 24 weeks is summarized in Table 2, demonstrating a gradual attenuation of the effect after 16 weeks while maintaining partial improvement at 24 weeks.

**Table 2:** Time-course changes in ankle circumference and soleus muscle thickness after botulinum toxin injection (*n* = 20).

Time point (weeks)	Ankle circumference (mean ± SD, cm)	Change from baseline (%)	Soleus thickness (mean ± SD, mm)	Change from baseline (%)
0 (Baseline)	22.8 ± 1.4	–	17.6 ± 1.2	–
4	21.5 ± 1.3	−5.7	15.8 ± 1.1	−10.2
8	21.2 ± 1.2	−7.0	15.0 ± 1.0	−14.8
12	21.1 ± 1.3	−7.4	14.9 ± 1.2	−15.3
16	21.5 ± 1.4	−5.7	15.6 ± 1.1	−11.4
24	21.8 ± 1.5	−4.4	16.0 ± 1.3	−9.1

Values are presented as mean ± standard deviation.

## Discussion

### Treatment outcomes and literature review

In this paper, we discuss three cases in which ultrasound-guided botulinum toxin injections successfully improved ankle contours in patients with thick ankles. Eight weeks after the treatment, average ankle circumference had decreased by 7 %, accompanied by 15 % reduction in soleus muscle thickness proven by ultrasound. These findings are consistent with previous research on efficacy of botulinum toxin to reduce muscle volume.^17^^,^^18^ For example, Jung studied 30 patients who received botulinum toxin injections for ankle contouring and found that the average ankle circumference reduced from 23.2 cm before treatment to 21.9 cm at 2 months after the injection.^1^ Our 8-week results likewise suggest that botulinum toxin alone can effectively slim the ankle region.

Traditionally, surgical procedures like partial muscle resection or selective neurectomy have been employed to reduce calf muscle hypertrophy. However, such approaches are invasive and involve risks such as scarring, contracture, and unpredictable muscle weakness.^4^^,^^5^ In contrast, botulinum toxin-induced muscle atrophy offers a relatively safe, non-surgical approach with minimal disruption to daily activities.^6^ Yet, the effects of botulinum toxin are not permanent; the muscle atrophy induced by the toxin usually lasts for only a few months and gradually declines thereafter. Around 5 to 6 months after treatment, muscle function tends to recover, leading to a partial return of the ankle’s previous circumference.^19^ Although the follow-up period in this case report was limited to 2 months, preventing an assessment of long-term progression, existing literature suggests that additional treatment may be required within 6 months to maintain results.^1^ The observed reduction in circumference (∼7 %) and soleus thickness (∼15 %) translated into visibly improved ankle contour and high patient satisfaction without functional compromise. However, given the temporary nature of botulinum toxin–induced atrophy, maintenance treatments may be necessary to sustain these aesthetic improvements. Future studies should incorporate longer observation periods to evaluate the need for repeat injections, optimal intervals, and any cumulative benefits over time.

### Advantages of ultrasound-guided injection

To maximize therapeutic effects of botulinum toxin and minimize side effects, injection site has to be precisely located.^20^ Traditionally, physicians relied on anatomical landmarks or palpation to correctly inject medications, but quality of these methods rely on the physicians’ experience. On contrary, US-guided injection allows us real-time visualization of the relevant muscles and nearby structures to overcome many of the limitations of blind approach.^21^ Physicians can easily find soleus muscle which lies deep in calf and is not easily palpated by hands. Physicians can guide the needle directly into the intended muscle by distinguishing the Achilles tendon from the soleus with ultrasound guidance. The approach enhances both the precision of toxin delivery and overall safety.^22^ In our cases, real-time ultrasound imaging helped us avoid blood vessels and other vulnerable structures, enabling safe and effective procedures without notable bleeding, nerve injury, or infection.

Moreover, nerve distribution and muscular arrangement should be considered when selecting injection sites.^23^ The motor nerve terminals of the soleus are mainly concentrated in the middle segment of the calf, making this region ideal for inducing muscle relaxation and atrophy via botulinum toxin.^13^^,^^24^ By contrast, focusing the injection solely on the gastrocnemius can lead to compensatory hypertrophy of the deeper soleus, potentially limiting the degree of circumference reduction.^13^ Taking these factors into account, our approach targeted the distal portion of the soleus, instead of the midsection, which was identified as the principal contributor to ankle circumference. The approach succeeded in achieving a more refined ankle contour both aesthetically and functionally.

### Limitations and future directions

Although the study included an increased number of patients and follow-up, it remains a single-arm, unblinded design. Therefore, potential observer and participant bias cannot be completely excluded. Future randomized controlled trials (RCTs) with control or sham groups are warranted to further validate both objective and patient-reported outcomes.

This report is limited by its very small sample size of only three cases, conducted at a single center. Additionally, the follow-up period of 2 months was relatively short, making it impossible to fully assess the long-term efficacy of botulinum toxin type A, potential cumulative effects of repeated injections, and changes in patient satisfaction over time. Future research should involve more patients and compare different types of toxins, including type B, to evaluate relative efficacy and safety. Systematic investigations into the impact of ultrasound guidance, optimal injection dosages, and intervals would also greatly aid in standardizing this procedure for ankle contouring. Finally, it is important to consider factors beyond muscular hypertrophy, such as subcutaneous fat and skeletal structure, that affect ankle circumference. Because botulinum toxin acts solely on muscle, it has limited effect when ankle thickness primarily stems from adipose tissue or bone configuration.^25^^,^^26^ Therefore, accurately determining whether muscle hypertrophy is the main cause of a patient’s thick ankles is crucial, as this helps clarify treatment suitability and manage patient expectations.

The duration of effect observed in this study (approximately 4–6 months) is comparable to other botulinum toxin applications, such as calf reduction or facial contouring, which also show attenuation within a similar timeframe. Repeated injections may reproduce consistent results without cumulative adverse effects.

In summary, ultrasound-guided botulinum toxin injections provide a safe and effective method of reducing excess muscle volume around the ankle and improve aesthetical muscular contour. Because the procedure is minimally invasive, it can reduce ankle circumference without scar. When carried out with proper dosage and technique, the method maintains high levels of patient satisfaction without hindering daily activities. Although this case report is based on a limited number of examples, further studies on the optimal use of botulinum toxin type A and its long-term efficacy may establish it as a robust option for shaping the ankle and lower leg.

## Funding

The authors have no financial interest to declare.

## Ethical approval

Not applicable. This study was conducted in compliance with the principles of the Declaration of Helsinki.

## STROBE compliance statement

This study was conducted in accordance with the STROBE (Strengthening the Reporting of Observational Studies in Epidemiology) guidelines for observational studies. A completed STROBE checklist has been submitted as supplementary material.

## Declaration of competing interest

None.
